# Looking for Discriminating Is Different from Looking for Looking’s Sake

**DOI:** 10.1371/journal.pone.0045445

**Published:** 2012-09-25

**Authors:** Hans-Joachim Bieg, Jean-Pierre Bresciani, Heinrich H. Bülthoff, Lewis L. Chuang

**Affiliations:** 1 Max Planck Institute for Biological Cybernetics, Department of Human Perception, Cognition, and Action, Tübingen, Germany; 2 Laboratoire de Psychologie et Neurocognition, CNRS, UMR 5105, Université Pierre Mendes France, Grenoble, France; 3 Department of Medicine, University of Fribourg, Fribourg, Switzerland; 4 Department of Brain and Cognitive Engineering, Korea University, Seoul, Korea; University of Muenster, Germany

## Abstract

Recent studies provide evidence for task-specific influences on saccadic eye movements. For instance, saccades exhibit higher peak velocity when the task requires coordinating eye and hand movements. The current study shows that the need to process task-relevant visual information at the saccade endpoint can be, in itself, sufficient to cause such effects. In this study, participants performed a visual discrimination task which required a saccade for successful completion. We compared the characteristics of these task-related saccades to those of classical target-elicited saccades, which required participants to fixate a visual target without performing a discrimination task. The results show that task-related saccades are faster and initiated earlier than target-elicited saccades. Differences between both saccade types are also noted in their saccade reaction time distributions and their main sequences, i.e., the relationship between saccade velocity, duration, and amplitude.

## Introduction

*Saccades* are rapid eye movements which are performed 3–4 times a second to fixate on a different spot in the environment [1]. The characteristics of saccades, notably *target-elicited saccades*, which follow the onset of a visual stimulus, have been thoroughly investigated. Past research has explored how visual properties of the saccade target, for instance, its luminance, color, or spatial arrangement, influence saccade planning and execution. For example, brighter stimuli lead to quicker initiation of saccades [2]. In experiments such as this, the saccade is elicited by the appearing target but the task does not inherently require the participant to fixate. This contrasts with the situation outside the laboratory. Here, saccades redirect the fovea, the region with highest visual acuity on the retina, to perform specific visual tasks [3]. The purpose of this paper is to compare the characteristics of classical *target-elicited saccades*, which do not require fixation per se, to *task-related saccades*, which require fixation due to task demands. Considering this distinction is important to avoid potential confounds in experimental tests of the oculomtor system’s variability.

The functional variability of saccade properties has been the topic of previous work, in particular work related to visually guided motor actions. Visual information is critical for accurate grasping and pointing [4]–[7]. The need to coordinate eye and hand movements could therefore be one factor that influences saccade characteristics. To test this hypothesis, Epelboim et al. [8] measured differences in saccade velocities across two conditions. One condition required participants to fixate a sequence of targets and the other to tap on them with a finger. Tapping resulted in faster saccades and a change in the relationship between saccade velocity, duration, and amplitude. This relationship, which is referred to as the saccadic *main sequence*, was thought to be the stereotypical result of brainstem saccade generator mechanics [9]. The work by Epelboim and colleagues demonstrates that changes in the main sequence occur when participants are engaged in an oculomanual task such as pointing. In a similar study with monkeys, Snyder et al. [10] found higher peak velocities and shorter durations for saccades that accompany arm movements. Like Epelboim et al., Snyder and colleagues also report main sequence differences. Apart from changes in saccade velocity, other studies reported differences in saccadic reaction time (RT), the time required to initiate a saccade following stimulus onset. For example, Lünenburger and colleagues [11], [12] found that saccades that support rapid pointing movements are initiated earlier than saccades that are made without such a movement to the target. In explaining their findings, Lünenburger et al. [11] suggested that saccade reaction times are adjusted to synchronize eye fixation so that foveal vision is provided during the final phase of the pointing movement.

This body of research suggests a functional role of saccade property adjustments due to the need to coordinate vision and hand movements. But are such adjustments only specific to oculomanual coordination? A study by Montagnini and Chelazzi [13] casts doubt on this assumption. In their study participants were not engaged in an oculomanual task but were required to rapidly identify an alphabetic letter at the saccade endpoint. Their results show similar changes in saccade properties, namely a decrease in saccade reaction time and an increase in velocity, when participants performed the rapid identification task in comparison to a condition where they only looked at the targets in succession. Related to this is the finding that saccades can be altered by verbally instructing participants to either emphasize speed or accuracy [14]. A comparison of differences in saccade RT distributions that were observed in this study with those observed in the study by Montagnini and Chelazzi suggests that the underlying process that leads to the reduction of RTs when performing an identification task could be different from the process that leads to the RT reduction when participants receive verbal instruction to emphasize speed over accuracy [13]. Instead of assuming a general effect of time pressure as it might be induced by verbal instructions, Montagnini and Chelazzi [13] therefore explained their findings on the grounds of *perceptual urgency*, i.e., as a natural response of the oculomotor system to stimuli that are only available very briefly.

The need to rapidly process visual information at the saccadic endpoint may cause changes in saccade properties [13]. This could also be an explanation for the results that were observed in the previously cited studies on oculomanual coordination. For example, differences in saccade characteristics in the studies by Epelboim et al. [8] and Snyder et al. [10] could stem from the need to perform two concurrent motor acts (eye and hand movements) or from the fact that movements of the eyes served a perceptual purpose in one but not in the other condition.

Two different studies provide additional support for the idea that saccades might show different characteristics if they are followed by a perceptual task. In experiments similar to that of Montagnini and Chelazzi [13] by Trottier and Pratt [15] and Guyader et al. [16], lower saccade RTs were measured for saccades that supported a visual discrimination task. It is important to note that, unlike in the study by Montagnini and Chelazzi [13], time pressure was not explicitly induced during these experiments.

The picture that emerges from this body of research suggests a general difference between classical target-elicited saccades and task-related saccades; a difference which might have confounded previous studies on motor coordination [8], [10]–[12] or time pressure [13]. To test this, the current study compared classical target elicited saccades to saccades that supported a visual discrimination task. Experiment 1 compared saccade RTs, peak velocity, duration, and gain in both types of saccades. Specifically, differences in the distribution of saccade RTs were measured. This made it possible to examine theoretical influences on saccade generation and enabled a comparison with the study by Montagnini and Chelazzi [13]. In experiment 2, saccade velocities and duration were measured across a range of amplitudes for both saccade types. This data was used to establish the velocity and duration main sequence. Under the premise of a general difference between task-related and target-elicited saccades, we expected a similar shift in main sequence curves as in the experiment by Epelboim and colleagues [8].

## Results

### Experiment 1: Looking vs. Discriminating

**Figure 1:**
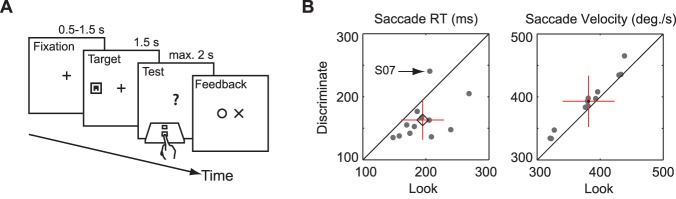
Experimental task and results (exp. 1). A. Schematic of the discriminate task. Participants fixated a central cross. This was followed by target onset either to the left or right of the fixation cross. Then, participants looked at the target and identified the location of the gap in the square. After this, the target disappeared and participants responded with the appropriate button press on a button box. Feedback was then presented depending on the response and actual gap location. The sequence of events was similar in the look condition except that no discrimination had to be carried out and participants were instructed to look at the target as quickly as possible. Here, participants confirmed trial completion by pressing the up button on the button box. Positive feedback was presented if a correct saccade was performed and the button response was given within the time window. B. Scatterplots of saccade properties with participant means, standard deviation (cross), and 95% confidence intervals (diamond) show shorter RTs and faster velocities in the discriminate condition. Data from participant S07 exhibits a potentially abnormal RT distribution (see text).

Saccade reaction time, peak velocity, duration, and gain were measured across two conditions of a saccade task. In condition 1 (discriminate condition) participants made a saccade to a target in order to identify it (see materials and methods section and Fig. 1). The target was a Landolt-square optotype, i.e., a small square with an opening on either the top or bottom (similar to [17]). Condition 2 (look condition) was identical to the discriminate condition, except that the square was shown without an opening. In this condition, participants were instructed to fixate the square as quickly as possible. Assuming that task-related saccades are categorically different from classical target-elicited saccades and that the results of previous experiments (e.g. [8]) can in part be explained by this difference, we expected shorter saccade reaction times and higher peak velocities in the discriminate condition.

Saccade RT and velocity were compared across the two conditions to assess whether saccades were faster and started earlier in the discriminate condition. Mean saccade reaction time in the look condition was 194 ms (SD 40 ms) compared to 163 ms (SD 32 ms) in the discriminate condition (individual means are shown in Fig. 1 B). This difference (95% confidence interval of difference: 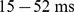, effect size 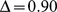) is statistically significant (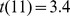, 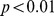).

Mean saccade peak velocity was 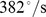 (SD 41) in the look condition and 393°/s (SD 41) in the discriminate condition. This difference (95% confidence interval of difference: 8–17°/s, 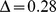) is statistically significant (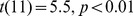).

A small but significant difference in saccade duration was found between both conditions. Mean saccade duration was 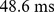 (SD 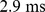) in the look condition and 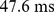 (SD 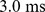) in the discriminate condition. This difference (95% confidence interval of difference: 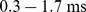, 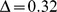) is statistically significant (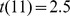, 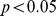).

Saccade velocity and duration are known to be functions of saccade amplitude. To test whether the increase in peak saccade velocity could be the result of different amplitudes, saccade gain was compared. In both conditions, saccade gain was close to one. In the look condition gain was 1.015 (SD 0.03) and 1.022 (SD 0.03) in the discriminate condition. This difference is not statistically significant (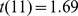, 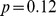).

**Figure 2:**
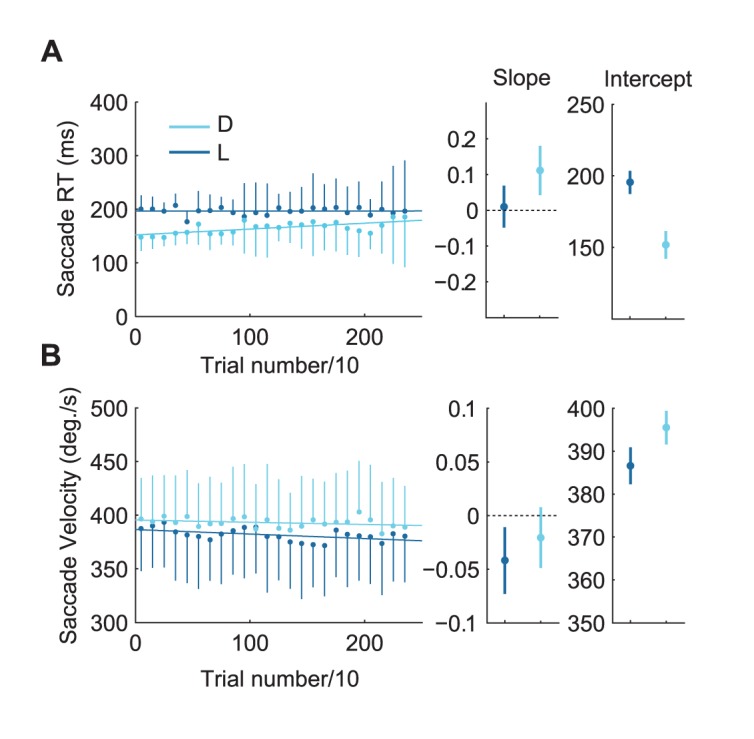
Changes in saccade parameters over time (exp. 1). Best linear fits across mean data. The data was binned in blocks of 10 trials. Data points show mean and variance for saccades performed in the look (L) and discriminate (D) condition. These trends suggest that the differences in saccade RT decreased over time while the difference in velocity increased.

To assess changes in saccade characteristics over the course of the experiment, best linear fits were obtained across trials (Fig. 2). This showed a positive correlation of saccade RT in the discriminate condition (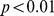, 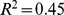) and a negative correlation of peak saccade velocity in the look condition (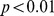, 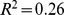), which could indicate that the difference in RT between both conditions decreased over the experiment while it increased for peak velocity.

**Figure 3:**
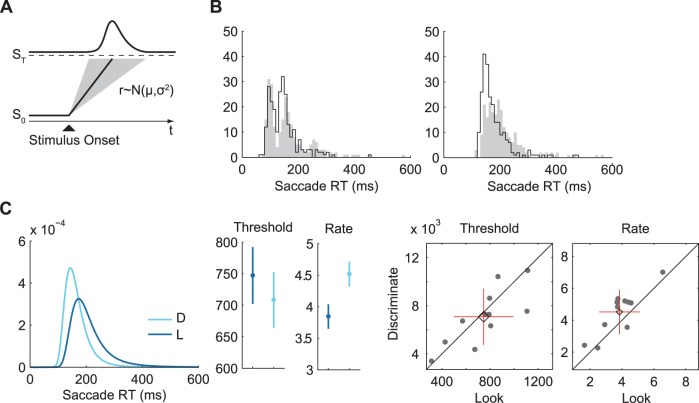
RT model, observed and theoretical RT distributions (exp. 1). A. Schematic of the LATER model. The model assumes that saccades are initiated once a decision signal rises from its baseline level 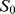 to a threshold 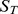 after target onset. The rate of rise 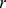 exhibits trial-to-trial variability, which is modeled by a normal distribution. The distribution of RTs resulting from this process is shown above. B. Observed RT distributions for two participants. Filled histograms show data for the look condition, outlines show data for the discriminate condition. Left: One of the observed bimodal distributions. For these, distribution parameters were estimated from the non-express part of the distribution (right mode). Right: Example for a more commonly observed unimodal distribution. C. Left: Theoretical RT distribution as predicted by the LATER model for RT data in the look (L) and discriminate (D) condition. Middle and right: Model parameters (threshold and rate) with 95% confidence intervals and scatterplots of the parameter distributions with mean, standard deviation (cross), and 95% confidence intervals (diamond) showing that the likely explanation for differences in the distributions is a change in the rate of rise.

A more thorough analysis of RT data was conducted to explain observable differences in RT distributions from raw RT histograms (Fig. 3 B). Sequential-sampling models such as the LATER model have been used in previous studies to successfully explain the shape of saccade RT distributions [18], [19]. The LATER model assumes that saccade initiation is determined by the accumulation of sensory evidence over time (Fig. 3 A). Specifically, it considers two main variables: a) the rate of rise of the decision signal [20] and b) the decision threshold [14], [19].

Maximum likelihood estimates of these variables were obtained on individual data and separately for each condition from the main part of RT distributions. Bimodal RT distributions were visible in the data of 2 participants, with the first mode around 100 ms, which is typically associated with express saccades [21], [22]. In line with previous research (e.g. [13]) parameters were fitted to the non-express part of the distribution in these datasets. Kolmogorov-Smirnov tests were carried out on each dataset to verify that reciprocal RT data was compatible with the assumption of a normal distribution, as predicted by the model. This was the case for all datasets (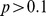) except for the data from one participant (participant S07, 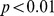). Inspection of this data showed an extreme spread of RTs in both conditions, which could be evidence for fatigue. The data of this participant was therefore excluded from further analyses (this was also the only dataset that exhibited longer RTs in the discriminate condition, see Fig. 1 B).

Average predicted distributions and parameter values are shown in Fig. 3 C. The theoretical distribution during the discriminate condition is characterized by a negative shift of the mode and decreased variability, which is evident from the shorter tail. Comparison of model parameters showed a significantly higher rate (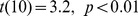, 95% confidence interval of difference: 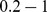, 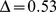) and only a small difference in threshold, which is not statistically significant (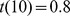, 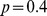). This suggests that the primary difference of RT data between both conditions was due to a change of the rate of rise of the decision signal, similar to previous findings which related changes in RT to a change in the rate of information supply [20] or effects of perceptual urgency [13].

Overall, the results clearly illustrate a fundamental difference between target-elicited and task-related saccades. In line with our hypothesis, task-related saccades exhibited shorter RTs and higher peak velocities. These findings are similar to those previously attributed to the effects of motor coordination [8], [10]. In addition, a comparison of saccade RT distributions using LATER model fits shows differences in the rate parameter – a finding which was previously attributed to effects of perceptual urgency [13].

### Experiment 2: Saccade Main Sequence

Saccade velocity and duration is strongly related to the amplitude of the required saccade. This relationship has been referred to as the saccade *main sequence* [9], [23]. Existing models explain this dependency as a result of duration-accuracy optimizations, which lead to optimal trajectories for any given target eccentricity [24], [25]. In addition, previous work suggests that saccade kinematics are also influenced by a variety of other aspects, for instance, the need to carry out an arm movement in coordination with an eye movement [8], [10].

Such modulations may not necessarily be the result of coordinated motor actions. The results from our first experiment suggest that task-related saccades in general, even in the absence of oculomanual actions, might have higher peak velocity than target-elicited saccades. In experiment 2, we extend this finding by examining saccade velocities across a range of amplitudes. With regard to the results of previous studies on motor coordination [8], [10], we expected main sequences of task-related saccades to show different properties (e.g., a steeper rise in velocity or higher saturation velocity) than target-elicited saccades.

To analyze changes in peak velocity across amplitudes, an exponential main sequence function of the form 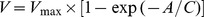 was fitted to individual peak velocity data [8], [23]. Here, 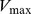 denotes the saturation velocity and 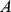 the saccade amplitude. The time constant 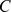 represents the amplitude at which 63% of the saturation velocity is reached and thus describes how quickly saturation is attained. Posterior amplitudes were used for fitting, i.e., the amplitudes that were actually performed, which were sometimes slightly longer or shorter than the required amplitudes.

**Figure 4:**
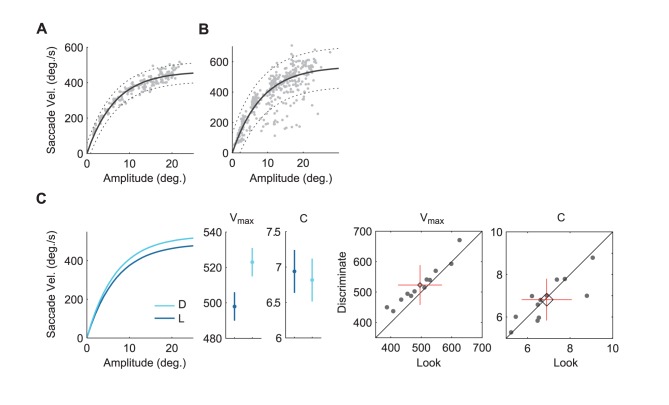
Saccade velocity main sequence (exp. 2). A. Example for a commonly observed distribution of saccade velocities as a function of amplitude. The figure shows the data for one participant in the look condition. The solid line shows the best fit of 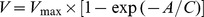. Dotted lines show 95% prediction intervals. B. The data from one participant in the look condition shows a significant number of datapoints below the main sequence curve (outside the prediction interval). This could indicate fatigue. C. Left: Theoretical main sequence curves for average parameters in the look (L) and discriminate (D) condition. Middle and right: Mean model parameters with 95% confidence intervals and scatterplots of the parameter distributions with mean, standard deviation (cross), and 95% confidence intervals (diamond). This shows a significant difference in saturation velocity 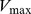.

Fig. 4 A shows a typical distribution of peak velocity data points and the resulting fit of the theoretical model (black line). Fig. 4 C shows the theoretical main sequences and parameters for both conditions following parameter averaging. On average, saccade duration was predicted best by 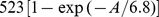 in the discriminate condition and by 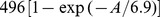 in the look condition. A statistical comparison of model parameters shows a significant difference in the saturation velocity 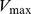 (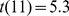, 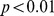, 95% confidence interval of difference: 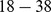, 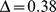) but not in the time constant 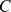 (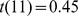, 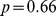).

A linear relationship between saccade duration and amplitude was assumed for saccades larger than four degrees [18], [26]. On average, saccade duration was predicted best by 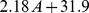 in the discriminate condition and by 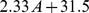 in the look condition. A comparison of parameter averages shows a significant difference in the slope parameter (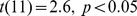, 95% confidence interval of difference: 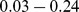, 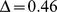) and an insignificant difference in the intercept parameter (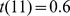, 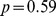).

An additional ad hoc analysis was performed for the data of participant S3, which showed a distinctive scatter of data points below the main sequence curve in the look condition. This resulted in a large difference in the time constant parameter (Fig. 4 B). Scatter below the main sequence curve is known to indicate fatigue [27]. To analyze this, we identified all data points outside a 95% prediction interval around the obtained main sequence. Further separation according to trial number showed that the majority of these outliers (34 of 37 points, 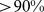) occurred in the second half of the experimental session (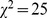, 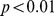). This suggests that, at least for this participant, fatigue due to repetitions might be one important factor which could explain the main sequence parameter differences, specifically, the difference in the saturation time constant. Overall, however, the results indicate that task-related saccades exhibit an increase in the saturation velocity of the velocity main sequence and a decrease in the slope of the duration main sequence in comparison to target-elicited saccades.

## Discussion

The present study compared the characteristics of *task-related saccades*, which supported a visual discrimination task, and classical *target-elicited saccades*, which were not followed by such a task. Experiment 1 showed that task-related saccades exhibit shorter reaction times, higher peak velocities, and shorter durations than target-elicited saccades. This is even more surprising since participants were instructed to perform target-elicited saccades as quickly as possible whereas emphasis was put on (task) accuracy when performing task-related saccades. The LATER sequential-sampling model [19] was used to model saccade RT distributions. An analysis of model fits revealed that differences between RT distributions of both saccade types could be explained by assuming a steeper rate of rise in the decision signal. Experiment 2 tested how the need to perform a discrimination task at the saccade endpoint affected the saccade main sequence, the relationship between saccade peak velocity, duration, and amplitude. Our results show an increase in the saturation velocity of the velocity main sequence and a decrease in the slope of the duration main sequence for task-related saccades.

Three basic explanations for the general differences in saccade RT and velocity can be excluded. First, it is well known that fundamental stimulus properties (e.g., luminance contrast) exert an influence on behavioral response characteristics and could have generated faster responses in one condition [2], [28]–[30]. Considering the small differences between the two targets, this explanation is unlikely. Second, the change in peak velocity could have been a concomitant of increased saccade gain. We dismiss this explanation by noting that the measured differences in gain were very small and not statistically significant. Third, an explanation in terms of dual-task effects on saccade RT, which were previously reported in saccade and discrimination tasks [31]–[33], is not applicable, since the location of the saccade target and discrimination target was not dissociated experimentally.

Previous studies which examined the functional variability of saccade properties obtained similar results, for example, higher saccade velocities and shorter reaction times during oculomanual actions such as pointing or grasping [8], [10] or object identification under time pressure [13]. How do these results relate to our findings and how can our findings be explained without invoking mechanisms of motor coordination or time pressure?

We speculate that differences between task-related and target-elicited saccades could be related to repetitions and motivation. Previous work has shown that massed repetitions of target-elicited saccades can result in a decrease in peak velocity [27], [34]–[37]. One explanation for our findings could therefore be that task-related and target-elicited saccades are affected differently by repetitions. Indeed, our results show a differential effect of repetitions on saccade characteristics, with peak velocity decreasing slightly in the look condition and saccade RT increasing in the discriminate condition (exp. 1). The work by Prsa et al. [37] shows that repeated saccades are not affected by muscular fatigue but by higher-order *mental fatigue*. In this respect, the current results could be explained by the interaction of two effects. First, a general arousal-related effect due to the monotonous nature of the task. Second, a more specific effect related to *motor readiness* due to motivational differences between the two saccade types [38]. A decline in arousal could have caused the general decrease in saccade velocity over time (exp. 1). In addition, presentation of negative feedback increases arousal [38]. This could explain why the decline in saccade velocity was less pronounced in the discriminate condition. Due to the presence of the discrimination task, more errors, and thus more negative feedback was presented in the discriminate condition compared to the look condition.

Previous writers have suggested that motivation influences saccade characteristics (e.g. [36]). Evidence has been provided, showing that saccade characteristics can be shaped by rewarding saccades [39]–[47]. In this regard, target-related saccades could be inherently more rewarding than classical target-elicited saccades. This could be the case because task-related saccades support completion of a meaningful task, which addresses competency-related needs [48], [49]. Following the argumentation of Chen-Harris et al. [36], this inherent reward value could decline with repeated stimulus presentations. This could explain the measured increase in RT over time in the current experiment, which was pronounced in the discrimination task. This explanation assumes that saccade characteristics can be affected by explicitly rewarding saccades, as well as by the reward value that is inherently associated with the task supported by the saccade. To the best of our knowledge, the existence of such an indirect influence is yet to be demonstrated and merits future investigation.

Differential motivational levels could also explain the obtained change in RT distributions which was revealed by the analysis using the LATER model [19]. This model predicts saccade RT on the grounds of a rising decision signal with a variable rate of rise and decision threshold (Fig. 3 A). A functional interpretation of the LATER model relates this decision signal to the accumulation of sensory evidence about the correct saccade choice. Evidence for this is provided by previous research, which found that manipulations of prior target probability and time pressure affect the baseline level or threshold of the hypothesized signal [14], [19]. Changes of the rate of rise of the decision signal were associated with the available amount of sensory information relevant for the decision, for instance, the coherence of dot movements in a random-dot kinematogram [20].

Best fits of the LATER model to the current data revealed that task-related saccade distributions exhibit a steeper rate of rise than target-elicited saccades. A change in the rate of rise was also observed by Montagnini and Chelazzi [13] in their comparison of saccade RTs to visual targets and saccades that were followed by a visual discrimination task under time pressure. This is incompatible with previous work by Reddi and Carpenter [14], which predicts that time pressure should lead to a change in the threshold parameter. Furthermore, Montagnini and colleagues showed that a gradual increase in time pressure did not result in a gradual decrease in saccade RT (see [13], experiment 2). Together, this suggests that the results of Montagnini and Chelazzi may not primarily reflect the time pressure that was associated with the discrimination task but, similar to the results of our own study, a more general influence of the visual task which followed the saccades.

Previous studies related a change in the rate of rise of the decision signal to the rate at which information is supplied to the saccadic choice process [20]. Neither the results of Montagnini and Chelazzi [13] nor our own results can be explained on the grounds of an unbalanced supply of information since target onset was equally perceptible in both conditions. However, a possible explanation for the change in rate of rise in line with this interpretation of LATER’s parameters could be that participants were less *efficient* in using the available information in target-elicited saccades, as a result of motivational differences. For instance, parietal and frontal brain areas (e.g., lateral interparietal area or frontal eye fields), which are known to be implicated in saccade generation and are likely implementations of an internal decision mechanism, are also known to be affected by the magnitude of expected rewards [50]. This could be a partial explanation for the data observed by Montagnini and Chelazzi [13], instead or in addition to the assumed effect of perceptual urgency.

A comparison of task-related and target-elicited velocity main sequences shows a higher maximal velocity (saturation velocity) and a small difference in the saturation time constant. This observation is quite similar to that of Epelboim et al. [8], who observed higher saturation velocities when participants tapped rather than looked at targets in succession. Differences in main sequence curves due to fatigue can be expected to lead to slower saturation. This is exemplified by the data of one participant in our experiments (Fig. 4 B). These data show a large saturation time constant difference between the two conditions, primarily due to a distinctive scatter of data points below the main sequence curve in the look condition. This scatter is similar to the observations by Schmidt et al. [27] who measured a fatigued observer. We did not observe similar patterns in the other data sets nor a significant overall difference in the saturation time constant parameter in our data. It is therefore unlikely that the overall difference in saccade velocities reflects a difference in the level of fatigue. Instead, following our previous argument, the increase in saccade velocity which is evident from the comparison of saturation velocity parameters could reflect the increased strength of the saccade target signal (see also [10]). This signal could primarily be influenced by salience and motivation, rather than effects of oculomanual coordination as Epelboim and colleagues [8] assumed.

In conclusion, the present study highlights a fundamental difference between task-related and classical target-elicited saccades. Task-related saccades exhibit shorter reaction times and higher peak velocities. These differences are also evident in systematic changes in saccade RT distributions and the relationship between saccade velocity, duration, and amplitude (main sequence). The present experiments also show that previous task-specific explanations of differences between task-related and target-elicited saccades might be too narrow in scope. Further experimentation is required to test alternative explanations, for instance, ideas put forth by neurophysiological research, which indicates a modulation of saccade characteristics by motivational aspects of the task.

## Materials and Methods

Two experiments were run to compare classical target-elicited saccades (look condition) against task-related saccades, which were required for completing a discrimination task (discriminate condition). Experiment 1 was conducted to assess differences in saccade characteristics and differences in saccade RT distributions. Experiment 2 investigated changes in the saccade main sequence parameters following the presentation of targets at different eccentricities.

### Participants

12 participants took part in experiment 1 (8 male, 4 female, ages 24–37) and another 12 participants took part in experiment 2 (7 female, 4 male, ages 21–31). All participants had normal or corrected to normal vision. In accordance with the World Medical Association’s Declaration of Helsinki, written informed consent was obtained from all subjects prior to experimentation and the procedures of the experiment had been approved by the ethical committee of the University of Tübingen. Participants were paid 8 EUR per hour for taking part in the experiment.

### Materials

In both experiments, participants sat in an adjustable chair in front of a CRT monitor (Sony GDM-FW 900, 100 Hz refresh rate, resolution 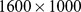) in a room with subdued light. A chin-rest provided support for the head at a viewing distance of 53 cm. An optical infrared head-mounted eye-tracking system was used to measure gaze at a sampling rate of 500 Hz (SR Research Eyelink II). A button box was used to collect manual responses. The eye-tracker and button box were connected to a dedicated computer which logged the data. Presentation of the experiment was controlled by custom-written software on a separate computer.

### Stimuli

Two types of visual targets were designed for the two conditions (look, discriminate). In the look condition, the target consisted of a 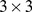 pixel block (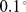 visual angle) with light gray color. In the discriminate condition, the same target was shown except that one pixel of the 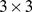 pixels was missing (corresponds to a gap of ca. 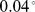 visual angle, 2.4 minutes of arc). The gap was located either at the top or bottom of the target. In both conditions, a white border was drawn around the target to make it discernible in the visual periphery.

A uniform gray background with a luminance of 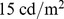 was shown throughout a trial. The target color was of a lighter gray with an average luminance of 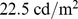, which corresponds to a Weber contrast of 0.5 (contrast was calculated as 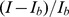 where 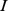 represents the stimulus intensity and 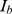 the background intensity). The target’s luminance contrast was adjusted separately for each participant to obtain a uniform degree of difficulty across participants. To do this, a block of trials of the discriminate condition was conducted at the beginning of the experiment. During this block of trials, the contrast was continuously adapted using the QUEST psychophysical procedure [51], so that similar difficulty levels were obtained for each participant (on average 86%, SD 9.6%, correct responses).

### Procedure

The basic experimental task required participants to make a saccade following target onset (Figure 1 A). Each trial commenced with the presentation of a fixation cross at the center of the computer screen. After a random delay with uniform distribution in the range of 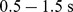, a target appeared at 9° eccentricity either to the left or right of the central fixation cross. The central cross stayed visible throughout the trial and the target remained visible for 1.5 s. This was sufficient time for participants to make a saccade to the target.

In the discriminate condition, participants were instructed to identify the opening of the target (top or bottom). Due to the small size of the gap, a saccade to the target was necessary in order to achieve this. After the target disappeared, participants indicated whether the target’s opening was at the top or bottom by pressing the corresponding button on the button box (up or down). In this condition, participants were told to identify the target as accurately as possible. No specific instruction was given with respect to saccade or response speed. In the look condition, participants were instructed to look at the target as quickly as possible. After the target disappeared, participants responded by pressing the up button on the button box to confirm trial completion and to keep the sequence of events consistent with the discriminate condition.

A similar procedure was used by two previous studies [15], [16]. However, it is not clear which role time pressure played in these experiments. In other experiments, time pressure was induced by an instructional emphasis on response speed [14] or limitation of target presentation time [13]. The former was also true for the study by Trottier et al. [15] and the latter applied to the work by Guyader et al. [16] (target presentation time 500 ms). We addressed these issues in our own experimental design. First, target presentation time was long enough (1.5 s) for participants to perform a saccade and still have sufficient time to discriminate the target. Second, target presentation and response input was separated into two phases of the trial. Participants first looked at the target. After that, the target disappeared and a question mark symbol prompted participants to press the appropriate response button. Early termination of a trial by participants through a premature response was therefore not possible.

Most everyday tasks consist of simple goal-directed behaviors [5], [52], [53]. Feedback on the results of an action is usually available in such behaviors [54]. For example, participants clearly perceived whether they successfully tapped on the targets in the pointing task presented by Epelboim et al. [8]. To provide clear feedback in the purely visual task that was used in the current experiment, a pictogram, which was either a circle for correct or cross for incorrect actions, was shown after each trial. In the discriminate condition, feedback was contingent on a participant’s response and the actual location of the opening. In the look condition, positive feedback was presented if a saccade to the target and the confirmatory button-press occurred within the respective time windows.

In total, each participant performed 480 trials in experiment 1. These comprised six blocks of 40 trials for each condition. The eye-tracking system was re-calibrated after each block. Regular 5 minute breaks were provided in intervals of three blocks of trials, during which the eye-tracker was removed. The order of conditions was fully counter-balanced between participants, half of which began a session with the discriminate or look condition. The entire experimental session lasted about 120 minutes.

The same procedure was also used in experiment 2 with the modification that targets were presented randomly at different eccentricities in the range of 1.5° to 20°. Since observers’ head motion was constrained by the eye-tracking equipment, we chose eccentricities close to those observed in natural gaze behavior [55], [56]. As a result, the eccentricities of these locations were smaller than those used by previous authors (using a head-free tracking system, Epelboim et al. [8] presented eccentricities up to 45°).

Four of the participants were tested in sessions which were held on successive days. Each experimental session lasted ca. 90–120 minutes. 900 data points were collected for these four participants. The remaining 8 participants were tested in single sessions lasting 120–160 minutes. During these sessions, conditions were presented randomly in blocks of 30 trials. This was done to minimize potential effects of day-to-day variability in performance due to different levels of fatigue. 360 data points were collected per participant during these recording sessions.

### Data analysis

Saccade detection was carried out by the Eyelink II system using a velocity (22°/s) and acceleration threshold (3800°/s^2^). The primary measures used to characterize saccadic eye movements were saccade reaction time (RT), peak velocity, duration, and gain. Saccade RT was defined as the time between the onset of the target and initiation of the movement. Saccade gain was defined as the size of the saccade divided by the step size, i.e., the distance between the location of gaze before the saccade and the target.

Data from the following trials were removed prior to the analysis: Trials with blinks during the critical time period shortly before or after the target onset, missed trials (no saccade or RT greater than 700 ms), anticipatory saccades (RT smaller than 50 ms), and inaccurate saccades with gains larger than 1.5 or smaller than 0.5.

In total 5760 data points were collected during experiment 1. 190 data points (3%) were removed due to application of the outlier rules. Analyses were carried out on the remaining data points. For saccade RT data, per-participant and condition cutoffs were employed [57]. These cutoffs removed data points 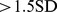 (median amount of points removed 7.5%, max. 13%). 6480 datapoints were collected in experiment 2. Of these, 460 data points (7%) were removed due to the outlier criterions.

If not indicated otherwise, paired two-tailed t-tests were employed for the comparison of mean differences (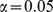) and mean-centering was performed for the computation of confidence intervals [58]. Becker’s 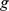, which is also known as Glass’s 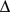 was used as a measure of effect size [59]. This is the mean difference between conditions divided by the baseline standard deviation (i.e., the SD of the look condition).
